# New Horizons of Artificial Intelligence in Medicine and Surgery

**DOI:** 10.3390/jcm13092532

**Published:** 2024-04-25

**Authors:** Valerii Luțenco, George Țocu, Mădălin Guliciuc, Monica Moraru, Iuliana Laura Candussi, Marius Dănilă, Verginia Luțenco, Florentin Dimofte, Oana Mariana Mihailov, Raul Mihailov

**Affiliations:** 1 Surgery I Clinic, Emergency Hospital “Sf. Ap. Andrei”, 800578 Galați, Romania; val050592@gmail.com (V.L.); raul.mihailov@ugal.ro (R.M.); 2 Faculty of Medicine and Pharmacy, “Dunărea de Jos” University of Galati, 800008 Galați, Romania; madalin.guliciuc@ugal.ro (M.G.); monica_ghionea@yahoo.com (M.M.); iuliana.candussi@ugal.ro (I.L.C.); marius_danilla@yahoo.com (M.D.); dimofteflorentin@yahoo.com (F.D.); 3 Clinical Children Emergency Hospital “Sf. Ioan”, 060011 Galați, Romania; verginia.lutenco@gmail.com

**Keywords:** artificial intelligence, medicine, surgery, machine learning, healthcare

## Abstract

**Background:** Ideas about Artificial intelligence appeared about half a century ago, but only now is it becoming an essential element of everyday life. The data provided are becoming a bigger pool and we need artificial intelligence that will help us with its superhuman powers. Its interaction with medicine is improving more and more, with medicine being a domain that continues to be perfected. **Materials and Methods:** The most important databases were used to perform this detailed search that addresses artificial intelligence in the medical and surgical fields. **Discussion:** Machine learning, deep learning, neural networks and computer vision are some of the mechanisms that are becoming a trend in healthcare worldwide. Developed countries such as Japan, France and Germany have already implemented artificial intelligence in their medical systems. The help it gives is in medical diagnosis, patient monitoring, personalized therapy and workflow optimization. Artificial intelligence will help surgeons to perfect their skills, to standardize techniques and to choose the best surgical techniques. **Conclusions:** The goal is to predict complications, reduce diagnostic times, diagnose complex pathologies, guide surgeons intraoperatively and reduce medical errors. We are at the beginning of this, and the potential is enormous, but we must not forget the impediments that may appear and slow down its implementation.

## 1. Introduction

Artificial intelligence is the capacity of a computer to imitate brain functions such as analysis, studying, planning and imagination. Some artificial intelligence technologies are over half a century old. Increased digital power, a large amount of electronic information and new processing technologies have led to the advancement of artificial intelligence in recent times. Artificial intelligence has become a main component of the digital revolution of humanity and has become one of the basic priorities of the European Union [1]. Often considered by people to be a field of computer science, artificial intelligence has broad applications, from arithmetic to information science, philosophy, biology, etc. Major changes will be brought by artificial intelligence applications, but even so, they have entered our everyday life without us feeling it. Artificial intelligence systems use techniques such as deep learning, machine learning and rules. Machine learning algorithms bring electronic information to artificial intelligence systems by using statistical techniques to approve artificial intelligence systems to learn. With automatic learning, artificial intelligence systems are becoming continuously more capable to give solutions without having to be especially programmed to carry this out. The utility in medicine could be enormous if all physicians tried to interact with artificial intelligence and understood that this is the future. But on the long road of adoption, there will certainly be many challenges that we have to face through a good implementation policy and correct ethics.

## 2. Materials and Methods

Our article was made with the aim of analyzing and discussing the relevant current knowledge regarding the involvement of artificial intelligence in medicine and surgery. We used the databases PubMed, Web of Science, Scopus and Medline to perform a detailed search using combinations of these specific terms: AI, artificial intelligence, deep learning, machine learning, healthcare, surgery and medicine. Due to the appearance of this fairly recent technology, we chose articles from 2015 to 2024, with the exception of four slightly older articles that helped us with some important information. However, we still relied on recent articles from the last 4 years. A total of 145 articles were identified. After reading of the titles, 93 were included. After reading of the summaries, 61 remained. In the end, we were left with 33 after reading the full articles. The most relevant articles, from our point of view, that addressed artificial intelligence in the medical and surgical fields helped us form a conclusion and carry out this original perspective article.

## 3. Discussion

### 3.1. Artificial Intelligence Includes Three Categories

1. Artificial narrow intelligence, also called “weak artificial intelligence”, is defined as the goal-oriented version. This kind of artificial intelligence is a reproduction of a human’s limited intelligence. Narrow artificial intelligence is fixed on achieving individual tasks but is limited to specific cases. “Siri (from Apple), Alexa (from Amazon), Google Search” are illustrations of narrow artificial intelligence.
2. Artificial general intelligence, also named “powerful artificial intelligence”, represents a type of artificial intelligence that can understand and learn all tasks just as a human would carry them out. The way of thinking, examining and solving for this artificial intelligence does not differ from that of a human being in some cases. Faced with an unfamiliar task, the artificial general intelligence system could find a solution.
3. Artificial superintelligence is considered the most advanced, powerful and intelligent type of artificial intelligence. Hypothetically, this level of intelligence not only performs and acts like human nature but will also become aware that it exists as an entity.

Artificial intelligence uses advanced algorithms to process information. It learns from data patterns or characteristics by combining large sets of information with appropriate algorithms. To understand how artificial intelligence actually works, its various subfields must be explained [2].

- Machine learning—trains a machine to make decisions by learning from past experiences. The main idea is that systems can learn with minimal human involvement and make different decisions. Supervised learning and unsupervised learning are two of the most popular machine learning methods. “Supervised machine learning” is based on labeled input and output information, while “unsupervised learning processes” in based on unlabeled or untreated data.
- “Deep learning”—imitates the functioning of the intellect in processing information and builds various models to then use in choosing solutions. “Deep learning” is a subgroup of “machine learning” in artificial intelligence. It can learn, unsupervised, through networks, from random information that has no structure. Deep learning is used in detecting volumetric bodies, understanding, converting sounds, translations and choosing solutions.
- “Neural networks” mimic human intelligence by containing a base of algorithms that try to recognize relationships in a data set. “Neural networks” resemble the connections between human brain cells—neurons having the same principles.
- “Natural language processing” is a function through which a machine can read, understand and interpret a language. The computer understands what the user communicates and can respond accordingly.
- “Computer vision” decomposes an image into thousands of parts and studies it in detail through certain visualization algorithms. Through this, the computer uses thousands of images, making associations, learning and giving results based on the accumulated experience.
- “Cognitive computing”, is that in which “Cognitive computing algorithms” imitate intelligence similar to a human being; analyze environments, sounds and images; and give answers. Cognitive computing, in theory, is an equal interaction between man and machine [3].

### 3.2. Current and Future Trends of Artificial Intelligence in Medicine

Artificial intelligence has already become an important pillar in medicine. Today’s reality already involves artificial intelligence in most subfields of human health. The initiation of artificial-intelligence hospital systems is one of the most crucial implements in medicine worldwide. Artificial intelligence technology will bring important changes to healthcare, becoming a necessity that more and more hospitals will need. It is already used in gastroenterology, dermatology, oncology, surgery, etc. Artificial intelligence also supports the development of new drugs, improvement of the quality of healthcare services and better streamlining of clinical workflow and vice versa, reducing costs for these services.

The initial diagnostic application of an artificial intelligence algorithm was approved in 2018 by the “Food and Drug Administration” in the USA—a program that automatically analyzes fundus images to assist in screening for diabetic retinopathy [4].

The adoption of artificial intelligence in healthcare continues to grow, with varied implications in pathological anatomy, imaging, cardiology and others, but it is important that medical personnel in every field interact with these technologies in order to provide safer, more cost-efficient and more productive medical care [5].

Globally, there has been a significant increase in the application of artificial intelligence in healthcare. Currently, some of the countries that are using artificial intelligence extensively in medicine include the United States of America, China, Japan, the United Kingdom, Germany and France. These countries have made significant progress in using artificial intelligence in an ample area of medical applications, such as medical data analysis, patient monitoring, medical diagnosis, personalized therapy and more. However, many other countries, including those in Central and Eastern Europe, are making efforts to implement artificial intelligence in their medical systems to increase the quality and performance of medical services [6].

In the first quarter of 2022, private firms invested billions of dollars in healthcare. One-third of this amount was allocated to one of the most promising fields in medicine—the integration and development of artificial intelligence [7].

Some models of using artificial intelligence in medicine:

- Medical diagnosis: Artificial intelligence can help identify symptoms and formulate diagnoses. Artificial intelligence technologies can be trained to recognize patterns and signs that indicate certain diseases, thus helping to make accurate and rapid diagnoses.
- Patient monitoring: Artificial intelligence can be used to monitor the conditions of patients and detect any changes in their health statuses: for example, in the intensive care unit or cardiology.
- Personalized therapy: Artificial intelligence can be used to create personalized therapies for patients based on their genetic profiles and medical histories. This can help choose the best treatments for patients, reducing the risks of side effects and increasing therapeutic efficiency.
- Optimizing workflow: Artificial intelligence can be used to optimize workflow in hospitals, reducing waiting time and increasing the efficiency of medical processes.

However, at the moment, the majority of medical personnel are skeptical about the integration of artificial intelligence in everyday practice. The technology should convince both the doctor and the patient to have full confidence and inspire its use on the ascending scale. Today, artificial intelligence performs well-defined tasks. It gives us binary answers: for example, present or absent and malignant or benign. An example would be tumor formation in dermatology or lamellae visualization in pathological anatomy [8]. 

Specialists are currently working to demonstrate the superior sensitivity and specificity of artificial intelligence to doctors. Another study currently under way is identifying target areas in radiotherapy. It seems that it will have satisfactory results, combining medical imaging with radiotherapy for precision that is not in the power of a doctor. But it is at the moment when the last decision is made by a specialist radiotherapist, and it will probably be many years before the paradigms change [9].

Another application of artificial intelligence would be in places that have limited medical resources. In areas with high prevalence of pulmonary tuberculosis, software analyzes lung radiological images and gives results with very high sensitivity and specificity [10].

A revelation of artificial intelligence could also be useful in diagnostic challenges and in diseases where the mortality rate is currently very high. Examples are sepsis and septic shock, where mortality reaches up to 50% and 63%, respectively: a rate that has not decreased too much in recent years [11].

**Figure 1:**
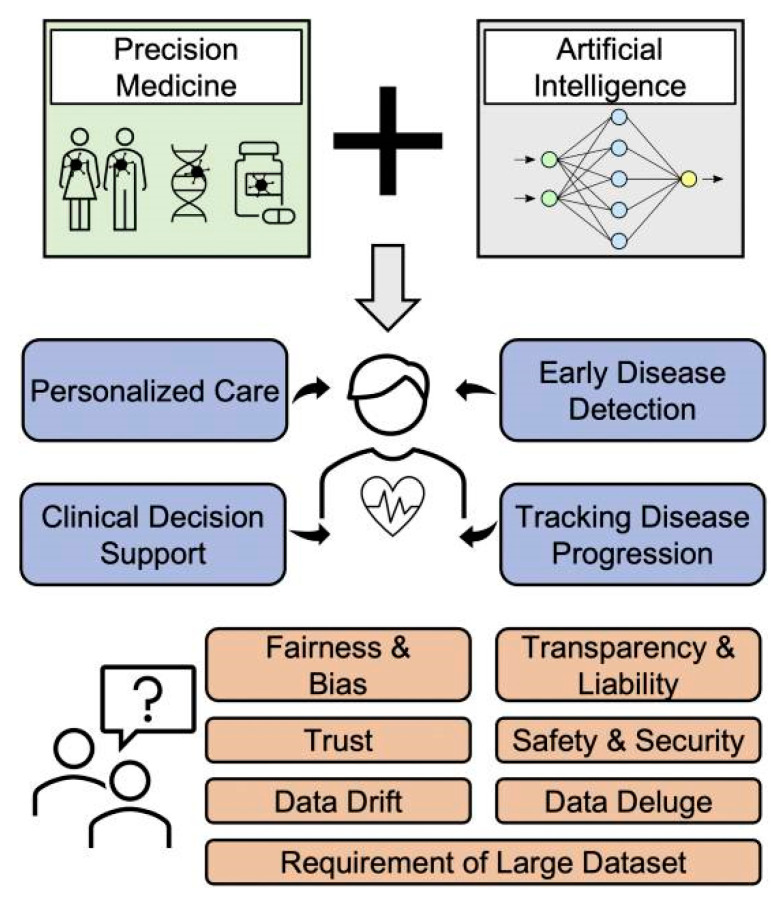
Overview of incorporation of artificial intelligence into precision medicine (reproduced from Ref. [13]).

Precision medicine has another advantage in artificial intelligence, where it has already started to make its mark and its evolution is at an accelerated pace (Figure 1). The utility is unlimited; artificial intelligence can make a synthesis of a patient’s electronic file with all biomarkers and associated pathologies and give the personalized medicament at the right time, which will also have an important contribution to preventive medicine [12]. 

### 3.3. Impediments and Ethical Dimensions

Even though huge progress has been made due to the integration of artificial intelligence in medical practice, many technical and ethical challenges await us in the future. These details could delay and hinder our trust and domain reliability. We can count a few examples. One of the important challenges that modern health management systems struggle with is the storage of large amounts of personal data and the transfer of these data. These data are used by artificial intelligence to give us answers to programmed tasks. Even though nowadays, there are multiple methods of encrypting sensitive data, there are still cases of data theft or breaches of confidentiality. It is clear that with more extensive development of technological involvement in medicine, there will be more involvement of hackers with cyberattacks. The goal is to improve data security and the adaptability of medical personnel to the new path that medical information will take [14]. Another impediment would be the limited availability of data. Artificial intelligence is not able to learn from small data sets, and often, there are insufficient data to train the learning models. This happens with quite rare diseases or with medical technologies that are still in the early phase of implementation [15,16], so erroneous data could appear. Machine learning models are very complex, with sophisticated algorithms. The transparency of a decision can, therefore, be questioned, and it will not be clear where and with whom the responsibility will be for artificial intelligence-assisted decisions. Biases can usually occur due to the data used for algorithm training or the inherent learning mechanisms of the algorithm. In addition, considering the field of healthcare, there could be additional biases due to the very complex mechanism between human relations and the decision-making process. These can be divided into those related to AI–doctor interactions (automation bias, feedback loop, rejection bias, allocation discrepancy) and AI–patient interactions (privilege bias, informed mistrust, agency bias) [17].

Finally, like other new technologies, artificial intelligence is subject to errors, so an initial mistrust could develop among the population and medical personnel, would be difficult to counteract and would decrease subsequent adoption.

Ethical issues are among the first problems that should be highlighted in the implementation of artificial intelligence in medicine. The theoretical framework should include these four pillars:

- Autonomy: A medical decision should be fully expressed by the freedom of the patient. He is free to be voluntary according to his personal ideas and principles.
- Benefit: Any action implemented on the patient must be for his benefit. You should take this into account.
- Non nocere: The primary principle is to not cause harm to the patient.
- Justice: This principle highlights equality, fairness and equity.

We are of the opinion that the doctors, patients and computer scientists who develop AI should take into account these four principles and any decision on an individual’s health should be taken to them accordingly [18].

### 3.4. Artificial Intelligence in Surgery

Surgeons spend a lot of time in the operating room to perfect their skills and analyze and participate in thousands of operations to learn different techniques and apply them in everyday practice, but human capabilities are limited. In this case, artificial intelligence comes to their aid, with the ability to absorb an enormous amount of information in seconds. By learning from thousands of different surgeries, artificial intelligence can help choose the best surgical techniques, giving both surgeons and patients better experiences. This can lead to standardized techniques with better results [19].

The advantages of using artificial intelligence are an increased diagnostic percentage of complex pathologies, reduced risk of medical errors and decreased time to diagnosis because what artificial intelligence carries out perfectly is building standardized assessment to remove personal subjective perception. Already, due to the results presented, it has been found that technologies based on artificial intelligence will be more and more advanced in the following times [20].

### 3.5. Preoperative Risk Prediction

It is well-known that with surgical intervention, there are some risks. According to statistics, around 20% of operations have simpler or more complex complications [21]. A patient will always want to know what risks he is subject to before surgical intervention. Risk prediction would greatly help both the decision to operate and the prediction of possible postoperative complications for the benefit of the patient.

Over time, various risk calculators and decision algorithms have appeared. Most of them address and prognosticate the “risk of major cardiac events”. This includes specimens like the “Revised Cardiac Risk Index (RCRI)” [22], “Perioperative Risk for Myocardial Infarction” or “Cardiac Arrest (MICA)” [23]. One of the most well-known risk calculators is the “American Society of Anesthesiologists score (ASA)”. It is often used in intensive care units, but it has a high degree of built-in subjectivity [24].

In 2013, the “American College of Surgeons National Surgical Quality Improvement Program (ACS-NSQIP)” published a “risk calculator”. It utilized data from 393 hospitals (1,500,000 subjects) to produce a “generalized linear mixed model to predict the risk of mortality and various complications”. This obtained great results [25].

In the past, all “risk calculators” applied traditional linear and additive models. The development of artificial intelligence has begun to use machine learning techniques, such as random forests, for example. The University of Florida has created “MySurgeryRisc”. Data from electronic medical records were used. After this app interacted with and received feedback from doctors, it managed to learn and adjust continuously [26]. Another prospective, nonrandomized pilot study of twenty physicians correlated the helpfulness and preciseness of preoperative risk estimation between doctors and “MySurgeryRisk”. It has been shown that

“Implementation of a validated, MySurgeryRisk computational algorithm for real-time predictive analytics with data derived from the electronic health records to augment physicians’ decision-making is feasible and accepted by physicians”.

[27]

Researchers at Duke University developed the Pythia risk calculator by extracting and managing data from large local institutions, using three machine learning methods: “logistic regression”, “random forest models” and “extreme gradient boosted trees”. The result was patterns with high prognostic performance. Patterns can be applied in daily work as a tool for the identification of patients with high risk, current assessment and treatment management [28].

Along with the evolution of artificial intelligence, prognostic scores have also evolved. In the future, doctors could use tools for objective and more exact prognosis of subject issues.

### 3.6. Diagnostics

One of the most important uses of artificial intelligence is the diagnosis of pathologies. Advances in imaging in recent years have provided so many data that only AI can process them.

An example would be the studies that tried to approach endoscopy for gastric cancer. Scientists have tried to apply artificial intelligence to aid in the endoscopic investigation of gastric malignancy because about 10% of upper gastrointestinal tumors are not identified by endoscopic examinations [29]. A convolutional neural network was applied to automatically recognize malign and benign zones under endoscopic exams, with an efficiency of 86–92.5%. The role of artificial intelligence in the endoscopy of gastric malignancy is not only to find it but also characterize it (ex: depth of wall invasion) [30,31,32].

Another study was performed on patients undergoing colorectal cancer screening or surveillance in eight centers (Italy, United Kingdom, United States of America). They underwent two consecutive colonoscopies on the same day, with and without artificial intelligence. In total, 230 subjects were admitted in that study research. The artificial intelligence achieved good results. The colorectal cancer omission rate halved. This pleads for the benefit of artificial intelligence in reducing failures in identifying small, subtle lesions in standard colonoscopy [33].

A study from Japan tried to diminish unnecessary resection. Its model, built with the aid of artificial intelligence, identified T1 colorectal tumors at risk for metastasis to lymph nodes. A machine learning artificial neural network was developed. This used information on subject age and sex, malignancy volume, affected area, histology type and grade and lymphatic and vascular invasion. The artificial neural network surpassed protocols in diagnosing subjects with T1 colorectal cancers with invasion in lymphatic nodes. This model can be helpful in identifying subjects who require supplementary surgery after colonoscopic T1 resection [34].

### 3.7. Intraoperative Applications

Computer vision is a computer science discipline that utilizes artificial intelligence techniques such as deep learning to process and analyze visual data. Artificial intelligence, through computer vision, enables PCs to understand viewable steps and connect with people in real time [35]. With enough exercise and the adding of a system of hundreds of surgical interventions, artificial intelligence can advise throughout surgery. To acquire images, laparoscopic cases are used because of the ease with which images are obtained. In the beginning, a lot of work was carried out for the standard stages.

1. *Recognition of the surgical phase*

Surgical phase (or stage) recognition is one of the most frequently investigated utilizations of machine learning in intraoperative video analysis. The surgical interventions have specific steps. The experience of surgeons can be utilized to train artificial intelligence and test the classification accuracy of neural networks [36].

2. *Recognition of instruments*

Surgical instrument recognition is another application. From the detection of a chosen instrument, one can obtain indications of the surgical stage and an analysis of the complexity of interventions.

3. *Gestures and error recognition*

The recognition of gestures and movements is a more complex phase that unites the other two previously mentioned. The deep learning tried to classify the participants according to skills in three levels of professionalism (beginner, intermediate and expert), so artificial intelligence can be a good evaluator of professional surgical skills [37].

4. *Recognition of anatomical landmarks*

A recent addition to machine learning in intraoperative applications is the capability to find surgical anatomy. Applying laparoscopic cholecystectomy procedures, Madani et al. tried to find and train artificial intelligence models that can differentiate safe and unsafe areas of dissection and recognize anatomical landmarks during a laparoscopy (cholecystectomy). Artificial intelligence models were developed on “2627 random frames from 290 laparoscopic videos”. They managed to demonstrate that technology can guide us in real time and reduce the risk of intraoperative errors. The efficiencies of identifying the safe and unsafe areas were 0.94 and 0.95, respectively [38].

Currently, the American Society of Gastrointestinal and Endoscopic Surgeons is making great efforts to convoke an international accord on guidelines for video images collected for analysis with machine learning and computer vision. Future phases will introduce the development of live-surgery “decision support”. As artificial intelligence accumulates more and more cases and intraoperative video images, it will have excellent surgical intelligence that will be able to assist any surgeon in any corner of the world with the best surgical care.

## 4. Conclusions

We wished to show that artificial intelligence is already involved and will increasingly intervene in medicine: more precisely, in the management of surgical cases. Artificial intelligence and its application are at the beginning of development, although important progress is being made with the artificial intelligence. This theme is in continuous exploration and progress, but at the same time, we must not forget the obstacles that can appear and make this evolution much more difficult. Ethical concepts are also of great importance, and until they are well-established, we will not progress quickly. As with any new technology, critical evaluation of new studies, software and equipment is necessary to adequately assess the impact on people’s care and surgical evaluation. However, in the present, the data on the potential applications of artificial intelligence in surgery have been promising. We think computers will not ever fully replace a physician or surgeon, but they can both singularly and collectively improve physician performance. 

## References

[1] Ebers M., Hoch V.R.S., Rosenkranz F., Ruschemeier H., Steinrötter B. (2021). The European Commission’s Proposal for an Artificial Intelligence Act—A Critical Assessment by Members of the Robotics and AI Law Society (RAILS). J.

[2] Mukhamediev R.I., Popova Y., Kuchin Y., Zaitseva E., Kalimoldayev A., Symagulov A., Levashenko V., Abdoldina F., Gopejenko V., Yakunin K. (2022). Review of Artificial Intelligence and Machine Learning Technologies: Classification, Restrictions, Opportunities and Challenges. Mathematics.

[3] Sarker I.H. (2022). AI-Based Modeling: Techniques, Applications and Research Issues Towards Automation, Intelligent and Smart Systems. SN Comput. Sci..

[4] U.S. Food & Drug Administration (2018). FDA permits marketing of artificial intelligence-based device to detect certain diabetes-related eye problems. Case Med. Res..

[5] Basu K., Sinha R., Ong A., Basu T. (2020). Artificial Intelligence: How is It Changing Medical Sciences and Its Future?. Indian J. Dematol..

[6] Chomutare T., Tejedor M., Svenning T.O., Marco-Ruiz L., Tayefi M., Lind K., Godtliebsen F., Moen A., Ismail L., Makhlysheva A. (2022). Artificial Intelligence Implementation in Healthcare: A Theory-Based Scoping Review of Barriers and Facilitators. Int. J. Environ. Res. Public Health.

[7] Shi M. (2022). Who Are the Most Active PE Investors in Healthcare? In: News & Analysis Driven by the PitchBook Platform. https://pitchbook.com/news/articles/most-active-pe-investors-healthcare.

[8] Buch V.H., Ahmed I., Maruthappu M. (2018). Artificial intelligence in medicine: Current trends and future possibilities. Br. J. Gen. Pract..

[9] Chu C., De Fauw J., Tomasev N., Paredes B.R., Hughes C., Ledsam J., Back T., Montgomery H., Rees G., Raine R. (2016). Applying machine learning to automated segmentation of head and neck tumour volumes and organs at risk on radiotherapy planning CT and MRI scans. F1000Research.

[10] Lakhani P., Sundaram B. (2017). Deep learning at chest radiography: Automated classification of pulmonary tuberculosis by using convolutional neural networks. Radiology.

[11] Tocu G., Mihailov R., Serban C., Stefanescu B.I., Tutunaru D., Firescu D. (2023). The Contribution of Procalcitonin, C-Reactive Protein and Interleukin-6 in the Diagnosis and Prognosis of Surgical Sepsis: An Observational and Statistical Study. J. Multidiscip. Healthc..

[12] Luțenco V., Rebegea L., Beznea A., Tocu G., Moraru M., Mihailov O.M., Ciuntu B.M., Luțenco V., Stanculea F.C., Mihailov R. (2024). Innovative Surgical Approaches That Improve Individual Outcomes in Advanced Breast Cancer. Int. J. Womens Health.

[13] Shandhi M.M.H., Dunn J.P. (2022). AI in medicine: Where are we now and where are we going?. Cell Rep. Med..

[14] Almalawi A., Khan A.I., Alsolami F., Abushark Y.B., Alfakeeh A.S. (2023). Managing Security of Healthcare Data for a Modern Healthcare System. Sensors.

[15] Ciuntu B.M., Tanevski A., Buescu D.O., Lutenco V., Mihailov R., Ciuntu M.S., Zuzu M.M., Vintila D., Zabara M., Trofin A. (2024). Endoscopic Vacuum-Assisted Closure (E-VAC) in Septic Shock from Perforated Duodenal Ulcers with Abscess Formations. J. Clin. Med..

[16] Şerban C., Constantin G.B., Firescu D., Rebegea L., Manole C.P., Truş C., Voicu D., Bîrlă R. (2020). Perforated Ileal GIST Associated with Meckel Diverticulum—A Rare Pathological Entity of Surgical Acute Abdomen. Chirurgia.

[17] Ueda D., Kakinuma T., Fujita S., Kamagata K., Fushimi Y., Ito R., Matsui Y., Nozaki T., Nakaura T., Fujima N. (2024). Fairness of artificial intelligence in healthcare: Review and recommendations. Jpn. J. Radiol..

[18] Solanki P., Grundy J., Hussain W. (2023). Operationalising ethics in artificial intelligence for healthcare: A framework for AI developers. AI Ethics.

[19] Hashimoto D.A., Ward T.M., Meireles O.R. (2020). The Role of Artificial Intelligence in Surgery. Adv. Surg..

[20] Sarno L., Neola D., Carbone L., Saccone G., Carlea A., Miceli M., Iorio G.G., Mappa I., Rizzo G., Girolamo R.D. (2023). Use of artificial intelligence in obstetrics: Not quite ready for prime time. Am. J. Obstet. Gynecol. MFM.

[21] Healey M.A., Shackford S.R., Osler T.M., Rogers F.B., Burns E. (2022). Complications in surgical patients. Arch. Surg..

[22] Bean M.G., Thompson A., Ghadimi K. (2018). Perioperative cardiovascular evaluation and management for noncardiac sugery. Essentials of Cardiac Anesthesia for Noncardiac Surgery: A Companion to Kaplan’s Cardiac Anesthesia.

[23] Peterson B., Ghahramani M., Harris S., Suchniak-Mussari K., Bedi G., Bulathsinghala C., Foy A. (2016). Usefulness of the Myocadial Infarction and Cardiac Arrest Calculator as a Discriminator of Adverse Cardiac Events after Elective Hip and Knee Surgery. Am. J. Cardiol..

[24] Wolters U., Wolf T., Stützer H., Schröder T. (1996). ASA classification and perioperative variables as predictors of postoperative oucome. Br. J. Anaesth..

[25] Bilimoria K.Y., Liu Y., Paruch J.L., Zhou L., Kmiecik T.E., Ko C.Y., Cohen M.E. (2013). Development and evaluation of the universal ACS NSQIP surgical risk calculator: A decsion aid and informed consent tool for patients and surgeons. J. Am. Coll. Surg..

[26] Bihorac A., Ozrazgat-Baslanti T., Ebadi A., Motaei A., Madkour M., Pardalos P.M., Lipori G., Hogan W.R., Efron P.A., Moore F. (2019). MySurgeryRisk: Development and Validation of a Machine-learning Risk Algorithm for Major Complications and Death after Surgery. Ann. Surg..

[27] Brennan M., Puri S., Ozrazgat-Baslanti T., Feng Z., Ruppert M., Hashemighouchani H., Momcilovic P., Li X., Wang D.Z., Bihorac A. (2019). Comparing clinical judgment with the *MySurgeryRisk* algorithm for preoperative risk assessment: A pilot usability study. Surgery.

[28] Corey K.M., Kashyap S., Lorenzi E., Lagoo-Deenadayalan S.A., Heller K., Whalen K., Balu S., Heflin M.T., McDonald S.R., Swaminathan M. (2018). Development and validation of machine learning models to identify high-risk surgical patients using automatically curated electronic health record data (Pythia): A retrospective, single-site study. PLoS Med..

[29] Menon S., Trudgill N. (2014). How commonly is upper gastrointestinal cancer missed at endoscopy? A meta-analysis. Endosc. Int. Open.

[30] Wu L., Zhou W., Wan X., Zhang J., Shen L., Hu S., Ding Q., Mu G., Yin A., Huang X. (2019). A deep neural network improves endoscopic detection of early gastric cancer without blind spots. Endoscopy.

[31] Hirasawa T., Aoyama K., Tanimoto T., Ishihara S., Shichijo S., Ozawa T., Ohnishi T., Fujishiro M., Matsuo K., Fujisaki J. (2018). Application of artificial intelligence using a convolutional neural network for detecting gastric cancer in endoscopic images. Gastric Cancer.

[32] Miyaki R., Yoshida S., Tanaka S., Kominami Y., Sanomura Y., Matsuo T., Oka S., Raytchev B., Tamaki T., Koide T. (2013). Quantitative identification of mucosal gastric cancer under magnifying endoscopy with flexible spectral imaging color enhancement. J. Gastroenterol. Hepatol..

[33] Wallace M.B., Sharma P., Bhandari P., East J., Antonelli G., Lorenzetti R., Vieth M., Speranza I., Spadaccini M., Desai M. (2022). Impact of Artificial Intelligence on Miss Rate of Colorectal Neoplasia. Gastroenterology.

[34] Kudo S.E., Ichimasa K., Villard B., Mori Y., Misawa M., Saito S., Hotta K., Saito Y., Matsuda T., Yamada K. (2021). Artificial Intelligence System to Determine Risk of T1 Colorectal Cancer Metastasis to Lymph Node. Gastroenterology.

[35] Xin F., Jiang Y., Yang X., Du M., Li X. (2019). Computer vision algorithms and hardware implementations: A survey. Integration.

[36] Kirtac K., Aydin N., Lavanchy J.L., Beldi G., Smit M., Woods M.S., Aspart F. (2022). Surgical Phase Recognition: From Public Datasets to Real-World Data. Appl. Sci..

[37] Van Amsterdam B., Clarkson M.J., Stoyanov D. (2021). Gesture Recognition in Robotic Surgery: A Review. IEEE Trans. Biomed. Eng..

[38] Madani A., Namazi B., Altieri M.S., Hashimoto D.A., Rivera A.M., Pucher P.H., Navarrete-Welton A., Sankaranarayanan G., Brunt L.M., Okrainec A. (2022). Artificial Intelligence for Intraoperative Guidance: Using Semantic Segmentation to Identify Surgical Anatomy during Laparoscopic Cholecystectomy. Ann. Surg..

